# Mental health and the global climate crisis

**DOI:** 10.1017/S2045796022000361

**Published:** 2022-12-02

**Authors:** Carlos Corvalan, Brandon Gray, Elena Villalobos Prats, Aderita Sena, Fahmy Hanna, Diarmid Campbell-Lendrum

**Affiliations:** 1 Department of Environment, Climate Change and Health, World Health Organization, Geneva, Switzerland; 2 School of Public Health, The University of Sydney, Sydney, Australia; 3 Department of Mental Health and Substance Use, World Health Organization, Geneva, Switzerland

**Keywords:** Community mental health, health outcomes, mental health, social environment

## Abstract

**Aims:**

Not only is nature essential for human existence, but many of its functions and contributions are irreplaceable. Studying the impact of these changes on individuals and communities, researchers and public health officials have largely focused on physical health. Our aim is to better understand how climate change also exacerbates many social and environmental risk factors for mental health and psychosocial problems, and can lead to emotional distress, the development of new mental health conditions and a worsening situation for people already living with these conditions.

**Methods:**

We considered all possible direct and indirect pathways by which climate change can affect mental health. We built a framework which includes climate change-related hazards, climate change-related global environmental threats, social and environmental exposure pathways, and vulnerability factors and inequalities to derive possible mental health and psychosocial outcomes.

**Results:**

We identified five approaches to address the mental health and psychosocial impacts of climate change which we suggest should be implemented with urgency: (1) integrate climate change considerations into policies and programmes for mental health, to better prepare for and respond to the climate crisis; (2) integrate mental health and psychosocial support within policies and programmes dealing with climate change and health; (3) build upon global commitments including the Sustainable Development Goals, the Paris Agreement and the Sendai Framework for Disaster Risk Reduction; (4) implement multisectoral and community-based approaches to reduce vulnerabilities and address the mental health and psychosocial impacts of climate change; and (5) address the large gaps that exist in funding both for mental health and for responding to the health impacts of climate change.

**Conclusions:**

There is growing evidence of the various mechanisms by which climate change is affecting mental health. Given the human impacts of climate change, mental health and psychosocial well-being need to be one of the main focuses of climate action. Therefore, countries need to dramatically accelerate their responses to climate change, including efforts to address its impacts on mental health and psychosocial well-being.

## Introduction

Climate change is a global crisis, its scale is already massive, and with inaction it continues to grow. It results in both acute hazards, such as hurricanes, floods and wildfires, and slower-onset threats, such as ecosystem changes, food and water insecurity and loss of place and culture. Climate change is increasingly having stronger and longer-lasting impacts on people, which can directly and indirectly affect their mental health and psychosocial well-being. The effects of unsustainable human activities, such as deforestation, ecosystem degradation and depletion and loss of biodiversity, and economies that are reliant on fossil fuels are leading to water and food insecurity, air pollution and contamination of land, rivers and oceans. All of these are having a measurable adverse impact on human health, mental health and well-being and further exacerbating the climate emergency. To address these concerns, the World Health Organization (WHO) issued a Policy Brief on mental health and climate change (WHO, 2022). This paper, based on this WHO report, aims to give visibility to this important topic and stimulate debate within the scientific community, and action by public health leaders.

Mental health conditions already represent a significant burden worldwide. Even without climate change, the situation for mental health globally is already challenging. In many countries large gaps exist between mental health needs and the services and systems available to address them. In fact, most people with mental disorders do not receive any care. This is particularly true in low- and middle-income countries, where fewer than 20% report receiving adequate services (Thornicroft *et al*., 2017). Other recent statistics add to these concerns: around 25% of years lived with disability are caused by mental (14.6%), neurological (7.6%) and substance use (2.7%) disorders (IHME, 2021); the median number of mental health workers for every 1 00 000 persons is low at only 13 (WHO, 2021a); around 1 billion persons worldwide are living with a mental disorder (IHME, 2021); the annual cost of common mental disorders is around $1 trillion (Chisholm *et al*., 2016); only 2% of Governments health budgets are spent on mental health (WHO, 2021a). These figures will likely be exacerbated by the climate crisis.

Not enough attention has been paid to mental health and psychosocial well-being in climate change literature, with studies on the topic emerging only since 2007 (Charlson *et al*., 2021). The connections between climate change and mental health and psychosocial well-being have been discussed mostly within the health frameworks of emergency and disaster management, particularly in the context of extreme weather events (WHO, 2019; IASC, 2021a). However, knowledge on the topic is growing (Augustinavicius *et al*., 2021; Charlson *et al*., 2021) and strong arguments can be made for expanding this focus beyond these frameworks to recognise the role of MHPSS within broader climate actions. Thus, although there are gaps in understanding the impact of climate change on mental health and psychosocial well-being, current knowledge is sufficient to act.

## The pathways by which climate change can affect people's mental health and psychosocial well-being are multiple

**Fig. 1.**
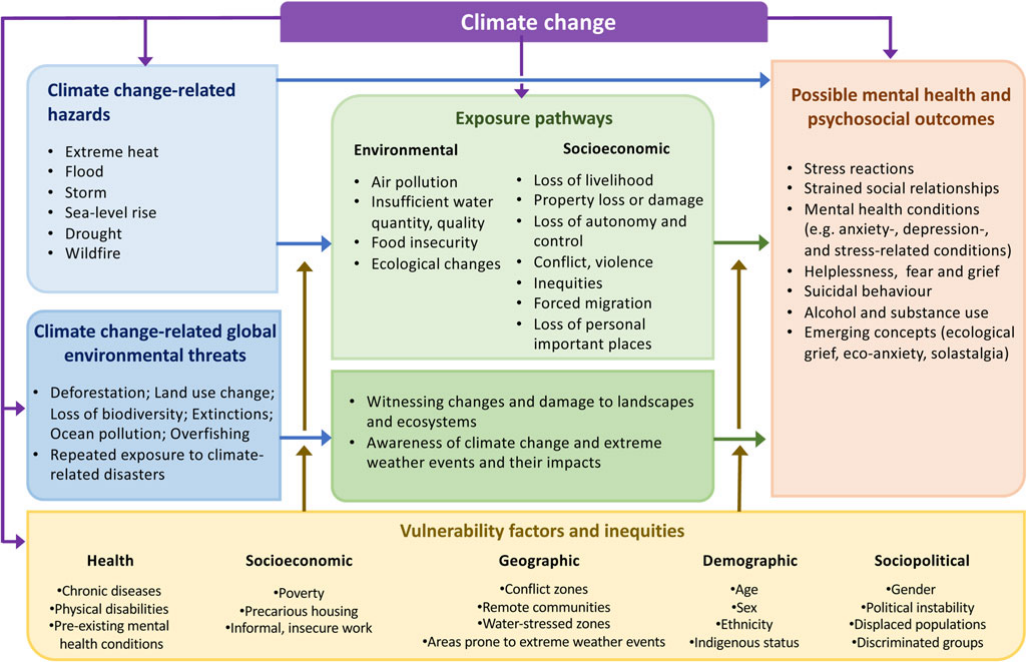
Main interlinkages between climate change and mental health.

The direct and indirect pathways by which climate-related hazards, long-term risks, exposure pathways and vulnerabilities interrelate to impact mental health are described in Fig. 1. These factors do not act in isolation. Instead, hazards may overlap (e.g. cascading events such as storms followed by floods). People may be exposed simultaneously to contaminated water and food insecurity while also being exposed to mosquito breeding sites. Existing population vulnerabilities may be exacerbated by climate hazards and long-term climate risks, resulting in aggravated inequities (WHO, 2021b). The resulting effects have considerable implications for mental health and well-being (examples in Box 1).

Box 1. Examples of mental health impacts of climate change and exposure pathways

**Table:** 

**Examples of mental health impacts** *Stress reactions* Climate-related hazards can lead to intense emotional suffering (Carroll *et al*., 2009; Bourque and Cunsolo Willox, 2014; Clayton *et al*., 2017; Cianconi *et al*., 2020). Most people experience some form of distress after an emergency but can effectively cope once basic needs are met and security and safety are restored (Bell *et al*., 2016; Clayton *et al*., 2017).
*Stress-related physical health problems* Stress can result in lower immune system responses, increasing vulnerability to air pollution and water-borne diseases (Simpson *et al*., 2011; Alderman *et al*., 2012; Clayton *et al*., 2017). Chronic distress is linked to sleep disorders, which can influence physical illness or worsen mental health and psychosocial well-being (Morey *et al*., 2015; Clayton *et al*., 2017; Cianconi *et al*., 2020). Psychological stress can increase risks of developing cardiovascular and autoimmune diseases and potentially cancers (De Blois *et al*., 2015; Morey *et al*., 2015; Abate *et al*., 2020).
*Mental health conditions* The development of mental health conditions, including depressive, anxiety and stress-related conditions, has been reported following extreme weather events (Berry *et al*., 2010; Hayes and Poland, 2018).
*Strained social relationships* Climate-related hazards lead to strains on interpersonal relations and intimate partner violence (Simpson *et al*., 2011; Clayton and Manning, 2018; Hayes and Poland, 2018). Other psychosocial impacts include family separation and disconnection from social support systems (e.g., children having to be temporarily relocated and required to attend another school or miss school (Kousky, 2016).
*Helplessness, fear and grief* Witnessing the slow impacts of climate change unfold can lead to worries about the future, along with feelings of helplessness and distress (Albrecht *et al*., 2007; Clayton *et al*., 2017). Some people experience feelings of loss, helplessness and frustration because they feel unable to stop climate change or make a difference (Hayes and Poland 2018). Many young people report feeling impairing distress and a sense of betrayal and mistrust of government in the face of climate inaction (Hickman *et al*., 2021).
*Increased risk of suicidal behaviour* Risk of suicide may be higher among those who have experienced repetitive or severe hazards (Norris *et al*., 2002; Berry *et al*., 2010). Rising ambient temperatures have also been linked to increased suicide rates in many countries (Burke *et al*., 2018; Kim *et al*., 2019; Parks *et al*., 2020).
**Examples of exposure pathways** *Loss of personally important places* Climate change threatens the environment and local communities, which in turn can create feelings of loss for important places and a sense of desolation (Albrecht *et al*., 2007; Cianconi *et al*., 2020). Changes in the physical environment (Cunsolo Willox *et al*., 2012) and disruption to peoples' home environments can lead to emotional distress and disorientation (Berry *et al*., 2010; Hayes and Poland 2018). For example, when people lose their homes to rising sea levels (Asugeni *et al*., 2015) or when land becomes unsuitable for farming practices or unable to support food crops due to long-term drought (Fritze *et al*., 2008), those affected may experience emotional distress and a sense of helplessness (Vins *et al*., 2015; Hayes *et al*., 2018). The loss of home environment can create a sense of a loss of continuity and belonging (Adger *et al*., 2013; Asugeni *et al*., 2015) and of personal identity (Scannell and Gifford, 2017; Hayes and Poland, 2018).
*Loss of autonomy and control* Climate change impacts basic needs and services and affects people's sense of autonomy and control (Albrecht *et al*., 2007; Deci and Ryan, 2011) – for example, making mobility a challenge for older people and people with disabilities (Clayton *et al*., 2017).
*Pollution* Air pollution is a significant driver of climate change that has also been associated with increased risk of mental health conditions (Braithwaite *et al*., 2019), including for children following mothers' exposure to particulate matter during pregnancy (Chun *et al*., 2020). (Classification based on Clayton *et al*., 2017).

The Intergovernmental Panel on Climate Change (IPCC), in its 6th assessment report states, with very high confidence, that climate-related illnesses, premature deaths, malnutrition in all its forms and threats to mental health and wellbeing are increasing. It also identifies that, at the global level, health systems are poorly resourced, and their capacity to respond to climate change is weak, with mental health support being particularly inadequate (IPCC, 2022).

The environmental, social and economic determinants of mental health (identified as exposure pathways in Fig. 1) include air quality, water quantity and quality, food security and safety, income and livelihoods, ecosystem changes and a number of other social and economic pathways. They are all negatively affected by climate change. For example, air pollution during periods of high temperatures can cause respiratory diseases that increase demand for health care services, reduce mobility and the capacity to work, and can lead to mental health consequences that range from minimal stress and distress to the development of mental health conditions, particularly in low-income settings (Dodgen *et al*., 2016). The case of prolonged droughts demonstrates a clear example of the impacts of climate change on these determinants. Droughts significantly disrupt agricultural production and lead to loss of livelihood, leaving many communities in poverty, a factor clearly linked with many common mental disorders (Lund *et al*., 2010). Droughts can also lead to water scarcity and food insecurity, both of which can negatively impact mental health and increase the risk for mental health conditions (Stanke *et al*., 2013; Vins *et al*., 2015; Pourmotabbed *et al*., 2020), the latter of which is associated with developmental delays, mental health issues and neurological problems that can result from malnutrition (WHO, 2006a, 2006b). Both food and water scarcity can also further contribute to population displacement, which disrupts family relationships and can leave those displaced with fewer resources, services and social support in the new community, all of which exacerbate mental health risks (Vins *et al*., 2015; Cianconi *et al*., 2020). Attention to the influence of climate change on determinants of mental health such as these is crucial for both understanding the impact and for taking climate action.

Climate change may also lead to increased conflict, or aggravated conflict dynamics, particularly in regions dependent on agriculture (Koubi, 2019), and to forced migration for some and forced immobility in challenging environments for others (Wright *et al*., 2021). Inevitably, conflict negatively impacts mental health and well-being, with one in five persons exposed to it experiencing a mental health condition (Charlson *et al*., 2019) and countless others enduring distress in the face of adversity. Meanwhile, migration is also commonly viewed as a risk factor for mental health and psychosocial problems, though more research is needed with populations migrating for reasons other than conflict (Meyer *et al*., 2017).

Certain groups will be disproportionately at risk due to climate change, depending on existing vulnerabilities and inequalities. This is particularly true in low- and middle-income countries, despite the fact that such countries have historically emitted low levels of greenhouse gases (Berry *et al*., 2010, 2011, 2018; Bourque and Cunsolo Willox, 2014; Hayes and Poland, 2018; Palinkas and Wong, 2019). For instance, indigenous people may be more likely to define well-being in terms of harmony with natural environments, which are significantly disrupted by climate change. As a result, they may be more strongly affected by the loss of even small amounts of land or wildlife or by other climate-related impacts. Children and adolescents are also uniquely affected and can experience strong reactions in response to the scale of the crisis and the lack of action taken (Hickman *et al*., 2021).

However, vulnerability is context-dependent and understanding who is vulnerable and in what way requires targeted assessment to identify contextual factors (WHO, 2021c). Any single factor may not necessarily determine vulnerability. However, in the case of different vulnerability factors, often interacting, the effects are multiplied (Gamble *et al*., 2016; Hayes *et al*., 2018; Cianconi *et al*., 2020). For example, someone may be older in age, or of lower socioeconomic status, or living in a water-stressed zone or living with a chronic disease, but may not be as vulnerable as someone who experiences all these factors simultaneously.

## Mental health and climate change: emerging concepts and concerns

There have been increasing efforts to better understand the mental health impacts of climate change. Individuals and communities may experience many intense emotions in the face of a changing climate, including sadness, fear, despair, helplessness and grief. Various terms have emerged to describe these responses, particularly among youth affected by climate change, including climate change anxiety (Clayton and Manning, 2018), solastalgia (Albrecht *et al*., 2007), eco-anxiety (Cordial *et al*., 2012), environmental distress (Higginbotham *et al*., 2012), ecological grief (Cunsolo and Ellis, 2018) and climate-related psychological distress (Bourque and Cunsolo Willox, 2014). Further research is needed to better understand these concepts, including what risk factors predispose people to these experiences and whether specific prevention and response actions are necessary. In any case, it must be noted that many of these reactions may represent understandable and congruent responses to the scale of the crisis the world faces (Hickman *et al*., 2021). In any emergency, including the global climate crisis, the terminology used to describe mental health and psychosocial problems can either support or stigmatise those affected. Care should be taken to ensure the use of terminology that normalises reactions to difficult situations and reinforces people's abilities to overcome adversity, rather than assuming the need for clinical intervention for all or labelling everyone affected as ‘traumatised’.

The potential beneficial mental health outcomes resulting from engaging in climate action have been described (Augustinavicius *et al*., 2021), such as increased well-being resulting from actively coping with the situation through climate action (Lawrance *et al*., 2021). However, others have discussed the potential distress experienced by people when confronting the scale of the problem (Sanson and Bellemo, 2021), indicating that climate action can also be harmful for mental health and well-being in some cases. Further research is required to develop a clear understanding of how climate action may promote and protect mental health and wellbeing and how better mental health can support increased action to address climate change.

## Recommendations to MHPSS and climate change actors

Approaches to address the mental health and psychosocial impacts of climate change must be implemented with urgency. We propose five recommendations to MHPSS and climate change actors.

### Integrate climate change concerns into the policies and programmes dealing with mental health, including MHPSS to better prepare for and respond to the climate crisis

Climate-related emergencies are increasing in frequency and severity. Better preparedness and better disaster risk management (DRM) are essential to protect people's physical and mental health in the face of climate-related hazards. The IASC MHPSS Reference Group (RG) recently produced a technical note linking disaster risk reduction (DRR) and MHPSS (IASC, 2021a) to support the delivery of a priority set of actions to reduce suffering and improve mental health and psychosocial well-being across and within DRM activities. However, there are additional long-term climate change risks that DRM alone cannot address. Thus, although the approaches documented in this guidance can be useful in reducing risks posed by the climate crisis, much more is needed to specifically respond to climate change, beyond climate-related disasters.

As a cross-cutting topic (Sphere Handbook, 2018; IASC, 2020), MHPSS should be integrated more broadly into climate change strategies and plans aiming to strengthen climate resilience and/or to promote the co-benefits of prevention and mitigation actions in other sectors as well. Likewise, climate change should also be integrated into mental health strategies and plans, including MHPSS. For instance, mitigation actions undertaken in the most polluting sectors (e.g. transport and urban planning) also have the potential to leverage important mental health co-benefits (e.g. a reduction in depression associated with active transport – walking and cycling) (USDHHS, 2008), while climate change adaptation may promote mental health and well-being. The MHPSS field can greatly benefit from broader recognition of the totality of climate risks, both acute hazards and slower-onset impacts, and the integration of climate change adaptation and mitigation strategies to better address these impacts.

### Integrate MHPSS within policies and programmes dealing with climate change and health

Key strategies in any response to climate change are mitigation and adaptation. There are important co-benefits to be gained from actions that contribute to climate change mitigation. Interventions related to active transport, for instance, are positive for physical health and can be positive for mental health too (WHO, 2016). Transport can also be important for access to services and social interaction, which have positive effects on mental health (WHO, 2011). Urban design that is environmentally friendly can provide green spaces for communities, with mental health benefits and stress reduction in different settings (WHO, 2016).

Regarding adaptation interventions, WHO recommends a systematic approach to strengthening the climate resilience of health systems. This is outlined in the WHO Operational Framework for Building Climate Resilient Health Systems (WHO, 2015). Mental health considerations and MHPSS approaches should be integrated within this health systems strengthening approach to build resilient health and mental health systems (Table 1). Both for mitigation or adaptation strategies, indicators, metrics and monitoring mechanisms are required to better understand the linkages between climate change and mental health.

**Table 1.** Integrating mental health considerations with climate change actions: examples of integrated mental health and climate change actions

Objectives	Examples of joint mental health and climate change actions
Leadership and governance	
Governance	Integrating climate change and MHPSS considerations into main policies and strategies in health-determining sectors, for adaptation (e.g. drought management and food production) and mitigation (e.g. urban planning and transport) Facilitating conditions for community mobilisation in climate change adaptation and mitigation actions
Policy	Including MHPSS in national strategies on health and climate change, such as Health in National Adaptation Plans (HNAPs), as well as in other relevant climate change policies and plans (e.g. Nationally Determined Contributions (NDCs) and Long-Term Low-Emission Sustainable Strategies (LT-LEDS))
Cross-sectoral collaboration	Establishing a single cross-sectoral MHPSS coordination mechanism that includes representatives and decision-makers from all sectors Developing functional pathways between sectors dealing with climate action (both adaptation and mitigation) and MHPSS services
Health workforce	
Human resources	Assessing and projecting climate change-related workforce capacity requirements Developing the capacity of general health-care workers to understand the mental health and psychosocial impacts of climate change in order to provide basic psychosocial support to those affected Training health managers on the effective integration of MPHSS into their climate change and health plans and strategies Developing and implementing organisational approaches to prevent and manage problems of mental health and psychosocial well-being among staff and volunteers
Organisational capacity development	Developing capacity to provide basic mental health care for people living with mental, neurological and substance use (MNS) conditions at every health facility Building referral pathways among mental health providers, general health-care providers, community-based support and other services
Vulnerability, capacity and adaptation assessments	
Vulnerability, capacity and adaptation options	Establishing indicators and baselines and assessing climate sensitivity and future risks to mental health and psychosocial well-being in climate change and health vulnerability and adaptation assessments Using these assessments to analyse local vulnerabilities and capacities with community actors Developing effective interventions to prevent and address mental health impacts, based on identified risks, vulnerabilities and capacities
Integrated risk monitoring and early warning	
Integrated disease surveillance and early warnings	Mapping geographic and seasonal distribution of hazardous events Developing and testing early detection and warning systems in collaboration with people with disabilities, including psychosocial disabilities, and people with MNS conditions Establishing surveillance systems integrating mental health outcomes and climate/weather information (e.g. heat stress and mental health)
Monitoring and evaluation (M&E)	Identifying and including indicators of climate-related stressors, vulnerability, MHPSS emergency preparedness and response capacity Establishing monitoring and evaluation mechanisms to measure the effectiveness of MHPSS activities (IASC, 2021b)
Communications	Preparing a risk communication strategy for disseminating essential information on climate risks to mental health and well-being, including information on positive coping
Health and climate research	
Research agenda	Including MHPSS in the national research agenda on climate change and health Developing a better understanding of, and response to, emerging concepts around mental health outcomes related to climate change
Climate-resilient and sustainable technologies and infrastructure	
Resilience	Assessing the vulnerability of health-care facilities, including MNS services related to climate change Ensuring that every health facility has at least one person trained, and a system in place, to identify and provide care for people with mental health conditions
Environmental sustainability	Assessing the carbon/environmental footprint of health-care facilities to create climate resilience and ensure sustainable technologies and infrastructure Developing and implementing plans to strengthen environmental sustainability which maximises benefits for the mental health of staff and patients
Medical product	Ensuring that consideration of climate change impacts on certain pharmacological agents, such as psychotropic medications, is properly integrated into relevant treatment protocols
Management of environmental determinants of health	
Monitoring and regulation	Establishing integrated monitoring systems that allow the collection and analysis of data on environmental hazards, socioeconomic factors and mental health outcomes (e.g. drought or heat stress management) Revising and enforcing regulations on key environmental determinants of mental health and psychosocial wellbeing (e.g. air quality and safety of housing)
Climate-informed health programmes	
Health programming	Developing or revising medium- and long-term national mental health plans to consider capacities that may be stressed and thresholds that may be exceeded by climate change Integrating climate change into existing national programmes for mental health
Delivery of interventions	Analysing seasonal trends – using information gathered in climate change and mental health vulnerability assessment risk maps – to target resources and preventive measures for those most at risk
Emergency preparedness and management	
Inform policies and protocols	Including MHPSS in national DRR strategies and emergency preparedness and response plans
Disaster risk management	Integrating MHPSS into risk assessments for current and future exposure to climate-related hazards Integrating MHPSS in relation to risk reduction, preparedness and response into health sector emergency plans for extreme weather events Including people living with mental health conditions and disabilities in contingency and evacuation planning for climate-related events Establishing sustainable and community-based services to facilitate recovery from climate-related hazards
Climate and health financing	
Health-specific funding mechanisms	Including climate change considerations in mental health research and programme development proposals submitted to and funded by health funding mechanisms
Climate change funding streams	Including mental health considerations in climate change and health proposals submitted to climate change funding streams (e.g. Global Environment Facility (GEF), Adaptation Fund (AF) and Green Climate Fund (GCF))

### Build upon global commitments

Much can be achieved by integrating MHPSS in the context of climate change by supporting and building upon existing global agreements including the Sustainable Development Goals (SDGs), the Paris Agreement and the Sendai Framework for Disaster Risk Reduction.

The SDGs address mental health and psychosocial well-being in the context of reducing non-communicable diseases (UN-DESA 2021). Additionally, several goals and targets directly or indirectly contribute to achieving good mental health and psychosocial well-being – particularly SDG targets related to climate hazards, exposure pathways and vulnerabilities that determine mental health – and their achievement can contribute to reducing negative mental health outcomes.

The Paris Agreement, a legally binding international treaty, aims to limit global warming to below 2 °C, and preferably to below 1.5 °C (UN, 2015). It has been described as a health agreement (WHO, 2018) because of the range of positive health outcomes that would be achieved if it were fully implemented. Although mental health is not specifically mentioned, it is implicit in the agreement's pursuit of equity and the reduction of vulnerability factors. It calls on Parties, when taking action on climate change, to ‘consider their respective obligations on human rights, the right to health, the rights of indigenous peoples, local communities, migrants, children, persons with disabilities and people in vulnerable situations and the right to development, as well as gender equality, empowerment of women and intergenerational equity’ (UN, 2015). Full implementation of the agreement, by all countries, would greatly advance progress on mitigating both the climate crisis itself and its devastating impacts on mental health.

The Sendai Framework for Disaster Risk Reduction 2015–2030 was adopted by countries at the Third UN World Conference on Disaster Risk Reduction in Sendai, Japan, in 2015. The Framework asserts that many disasters are exacerbated by climate change, that disasters are increasing in frequency and intensity, and that they are hindering progress towards sustainable development (UNDDR, 2015). Despite MHPSS being only briefly mentioned in priority four of the Sendai Framework (which focuses on recovery from crises), strong arguments exist for integrating MHPSS within all areas of DRR (Galappatti and Richardson, 2016; Gray *et al*., 2020). Examples exist globally of effective MHPSS and DRR programmes in areas heavily affected by climate change (Gray *et al*., 2021). The WHO Health Emergency and Disaster Risk Management Framework (WHO, 2019) highlights MHPSS as a core component of DRM, while a recent IASC technical note outlines guidance to better support MHPSS and DRR integration in preparing for, responding to and recovering from hazards globally (IASC, 2021a, 2021b).

### Implement multisectoral and community-based approaches to reduce vulnerabilities and address the mental health and psychosocial impacts of climate change

As the global climate crisis increases in intensity, priority should be given to the development of resilience at the community level. Community-based approaches recognise people affected by emergencies as active participants and leaders in efforts to improve individual and collective mental health and well-being, rather than passive recipients of external aid or support. Community-based MHPSS approaches also emphasise the value of working with communities to build on existing formal and informal systems of care that encourage recovery and resilience (IASC, 2019). These approaches can and should also be applied to addressing the impacts of climate change, including through existing community-based climate change adaptation and mitigation efforts.

Though more research is needed on the impact of climate activism on mental health and well-being, engaging in action to address climate change itself is crucial. Government leaders, climate adaptation and mitigation actors and MHPSS professionals must come together to promote community-based climate action that builds resilience and addresses the root causes of the problem, such as community-led initiatives to reduce household air pollution (Moreno Ramirez et al., 2015), simultaneously addressing its impact on the crisis and on mental health. Youth are well placed to engage in advocacy on this front and have done so in many forums. However, they should not be expected to act alone, or be forced to do so in ways that may be harmful for their development, because of inaction. Everyone has a role to play in building collective resilience and confronting the crisis.

### Address the large gaps that exist in funding both for mental health and for responding to the health impacts of climate change

Regardless of climate change, the availability of mental health services is limited by gaps in funding and a lack of trained personnel. Estimates show that mental health receives less than 1% of international aid for health (Charlson *et al*., 2017). Moreover, governments spend just 2.1% of their health budgets on mental health (WHO, 2021a). However, the costs of mental health impacts are very large. Lost productivity resulting from depression and anxiety alone, two of the most common mental disorders, costs the global economy approximately US$ 1 trillion each year (Chisholm *et al*., 2016). There are also funding gaps for responding to the general health impacts of climate change. Promised support by developed countries to less developed countries has not materialised (Timperley, 2021). Moreover, less than 0.5% of international climate change adaptation financing has been directed to climate adaptation to protect health (WHO, 2021d) and the figure is much less for mental health and psychosocial well-being. However, there are important mental health co-benefits of both climate change adaptation and mitigation actions, which support the argument for better use of existing funds.

## Concluding remarks

We must increase the visibility of the problem: The mental health consequences of climate change have until recently been an invisible problem. However, there is growing evidence of the various mechanisms by which climate change is affecting mental health. Countries need to dramatically accelerate their responses to climate change, including efforts to address its impacts on mental health and psychosocial well-being.

We must continue the research and debate on this emerging problem: The systemic, global and potentially irreversible effects of the crisis have given rise to emerging concepts such as climate change anxiety, solastalgia, eco-anxiety and ecological grief. In many cases, these reactions may represent understandable and congruent responses to the crisis the world faces, and yet their impact can be significant. Although there is a need for further research, the world has sufficient experience and evidence to guide immediate action.

We must act now to protect mental health: Strengthening the link between mental health and climate change is an opportunity to create a more holistic and coordinated response. Effective interventions are available and can be implemented immediately. With further support from policy-makers, researchers and MHPSS and climate actors, other interventions will be developed and a holistic response can be implemented.

We must ensure mental health is included in the climate change agenda: Given the human impacts of climate change, mental health and psychosocial well-being need to be one of the main focuses of climate action. There needs to be a commitment both politically and financially and across all sectors to make MHPSS and climate action a priority. This is the only way to achieve justice for all those who are affected.
